# Effect of tea intake on genetic predisposition to gout and uric acid: a Mendelian randomization study

**DOI:** 10.3389/fendo.2023.1290731

**Published:** 2024-02-02

**Authors:** Yunfeng Yu, Xinyu Yang, Gang Hu, Keke Tong, Yuman Yin, Rong Yu

**Affiliations:** ^1^ Department of Endocrinology, The First Hospital of Hunan University of Chinese Medicine, Changsha, Hunan, China; ^2^ College of Chinese Medicine, Hunan University of Chinese Medicine, Changsha, Hunan, China; ^3^ Department of Gastroenterology, The Hospital of Hunan University of Traditional Chinese Medicine, Changde, Hunan, China

**Keywords:** tea intake, caffeine, gout, gout due to impairment of renal function, uric acid, Mendelian randomization

## Abstract

**Objective:**

The effect of tea on gout and uric acid is still controversial. This study aims to analyze the effect of tea intake on genetic predisposition to gout, idiopathic gout, gout due to impairment of renal function as well as uric acid by Mendelian randomization (MR).

**Methods:**

Forty independent single nucleotide polymorphisms (SNPs) associated with tea intake were selected from UK Biobank. SNPs for uric acid were obtained from BioBank Japan, SNPs for gout were obtained from UK Biobank, and SNPs for gout due to impairment of renal function and idiopathic gout were derived from FinnGen. The causal relationship of exposure-outcome was tested using inverse variance weighted, MR-Egger and weighted median. MR-Egger intercept was employed to assess horizontal pleiotropy, Cochran’s Q test was used to assess heterogeneity, and leave-one-out sensitivity analysis was utilized to analyze the stability of the results.

**Results:**

The results of MR analysis showed that tea intake was negatively associated with gout due to impairment of renal function (OR 0.997, 95% CI 0.994 to 0.999, *P* = 0.017), whereas there was no causal association with gout, idiopathic gout, and uric acid (*P* > 0.05), for which sensitivity analysis suggested that these results were robust.

**Conclusions:**

There was a genetic predisposition effect of increased tea intake on the reduced risk of gout due to impairment of renal function, whereas there was no such effect on gout, idiopathic gout, and uric acid. Tea intake may become an important option in the dietary treatment of gout due to impairment of renal function.

## 1 Introduction

Gout is an inflammatory arthritis caused by the precipitation of uric acid monosodium salt crystals in the joint space (1). Epidemiologic studies have shown that the prevalence of gout is 1-4% globally (2) and continues to show an increasing trend year after year (3). Pain with swelling in the joints is the main clinical symptom of gout patients and a common cause affecting their quality of life (4). In addition, gout is closely related to cardiovascular disease and kidney disease, and it is a significant cause of premature death in patients with cardiovascular disease (5). Abnormally elevated serum uric acid is a critical factor in the pathogenesis of gout (6), and uric acid-lowering therapy can effectively reduce the risk of gout attacks and reduce the formation of tophi (1). Uric acid is the end product of purine nucleotide metabolism (7), and about one-third of the purines in the body originate from food intake (8). Therefore, relevant guidelines developed by the American College of Rheumatology (ACR) and the European League Against Rheumatism (EULAR) have emphasized the importance of dietary interventions for gout (9, 10).

As a non-alcoholic beverage widely consumed worldwide (11), tea is rich in tea polyphenols, theanine, tea polysaccharides, and caffeine, which greatly benefit for the human body (12, 13). Existing studies have found that tea can assist in lowering lipids, weight loss, and improving glucose tolerance, and has potential benefits for diseases such as hypercholesterolemia, obesity, and metabolic syndrome (14, 15). However, the effect of tea on the risk of gout and uric acid is still controversial, and it remains to be further investigated. Mendelian randomization (MR), as a method of genome-wide association study (GWAS) data collation (16), uses genetic variants to construct instrumental variables of exposure to estimate the causal relationship between exposure variables and outcome variables (17). Because genotypes are assigned randomly, the association between genetic variants and outcome measures is not affected by confounding variables (18). Therefore, this study utilized MR to explore the effect of tea intake on genetic predisposition to uric acid, gout, gout due to impairment of renal function as well as idiopathic gout.

## 2 Materials and methods

### 2.1 Study design

**Figure 1:**
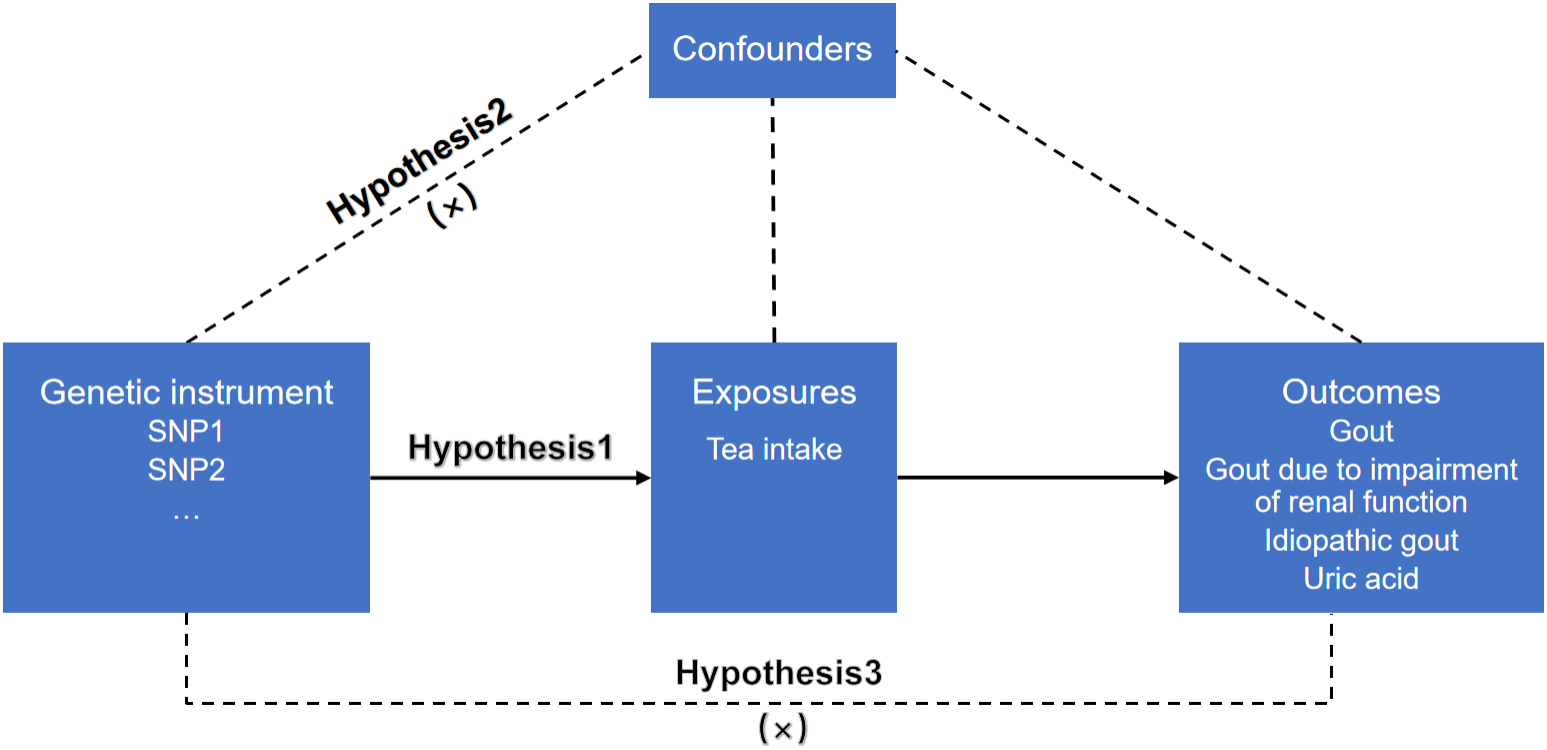
MR design for causal analysis of tea intake on genetic predisposition to gout and uric acid.

MR relies on three basic assumptions (19). 1) Association hypothesis: Single nucleotide polymorphisms (SNPs) are strongly associated with exposure variables. 2) Independence hypothesis: SNPs and confounding variables are independent of each other. 3) Exclusivity assumption: the SNPs cannot act on the outcome variables through pathways other than the exposure variables. SNPs are a common form of genetic variation in the genome that may affect gene expression or function, which in turn positively or negatively affecting an individual’s traits, disease susceptibility, and other aspects. SNPs with positive or negative effects on gout and uric acid were included in the outcome variables included in this study. The design flow of the MR is shown in **Figure 1**.

The diagnosis of gout and hyperuricemia is referenced below: 1) Gout: Monosodium urate crystals by polarising light microscopy of synovial fluid or tophaceous material (20). 2) Idiopathic gout: Gout of unknown origin. Monosodium urate crystals were identified in the synovial fluid or tophaceous material by polarising light microscopy, and the possibility of secondary gout was ruled out (20). 3) Gout due to impairment of renal function: Gout caused by decreased uric acid excretion due to renal insufficiency. Monosodium urate crystals were detected by polarising light microscopy in synovial fluid or tophaceous material and determined to result from renal impairment (21). 4) Hyperuricemia: Classically defined as a serum urate of more than 7.0 mg/dL in men or more than 6.0 mg/dL in women (22).

### 2.2 Data sources

Data on tea intake, gout, gout due to impairment of renal function, idiopathic gout, and uric acid were obtained through the publicly available BioBank Japan (biobankjp.org/en/), UK Biobank (www.nealelab.is/uk-biobank) and FinnGen (www.finngen.fi/fi). As all data were obtained from publicly available databases, no additional ethics approval was required.

### 2.3 Selection of genetic instrumental variables

The GWAS database includes GWAS of tea intake in 447,485 individuals of European ancestry. Firstly, the SNPs closely related to exposure variables were screened according to the *P*< 5×10^-8^ standard to satisfy hypothesis 1. Secondly, the criteria of R^2^< 0.001 and kb = 1 0,000 were used to continue screening SNPs to avoid potential bias caused by linkage disequilibrium (LD). Thirdly, the F-value of each SNP was calculated separately, and SNPs with F > 10 were used as instrumental variables in this study. F = [ $R^{2}$ /( $1-R^{2}$ )]*[ (N−K−1 )/ K ]. Among them, $\;R^{2}=2*\left(1-MAF\right)*MAF*\beta^{2}$ . R^2^: The cumulative explained variance of the selected IVs on exposure; MAF: the effect of minor allele frequency; β: estimated effect of SNP; N: sample size of the GWAS. Finally, referring to PhenoScanner (www.phenoscanner.medschl.cam.ac.uk) and related literature, SNPs potentially associated with uric acid or gout were removed to meet hypothesis 2.

### 2.4 Data analysis

**Figure 2:**
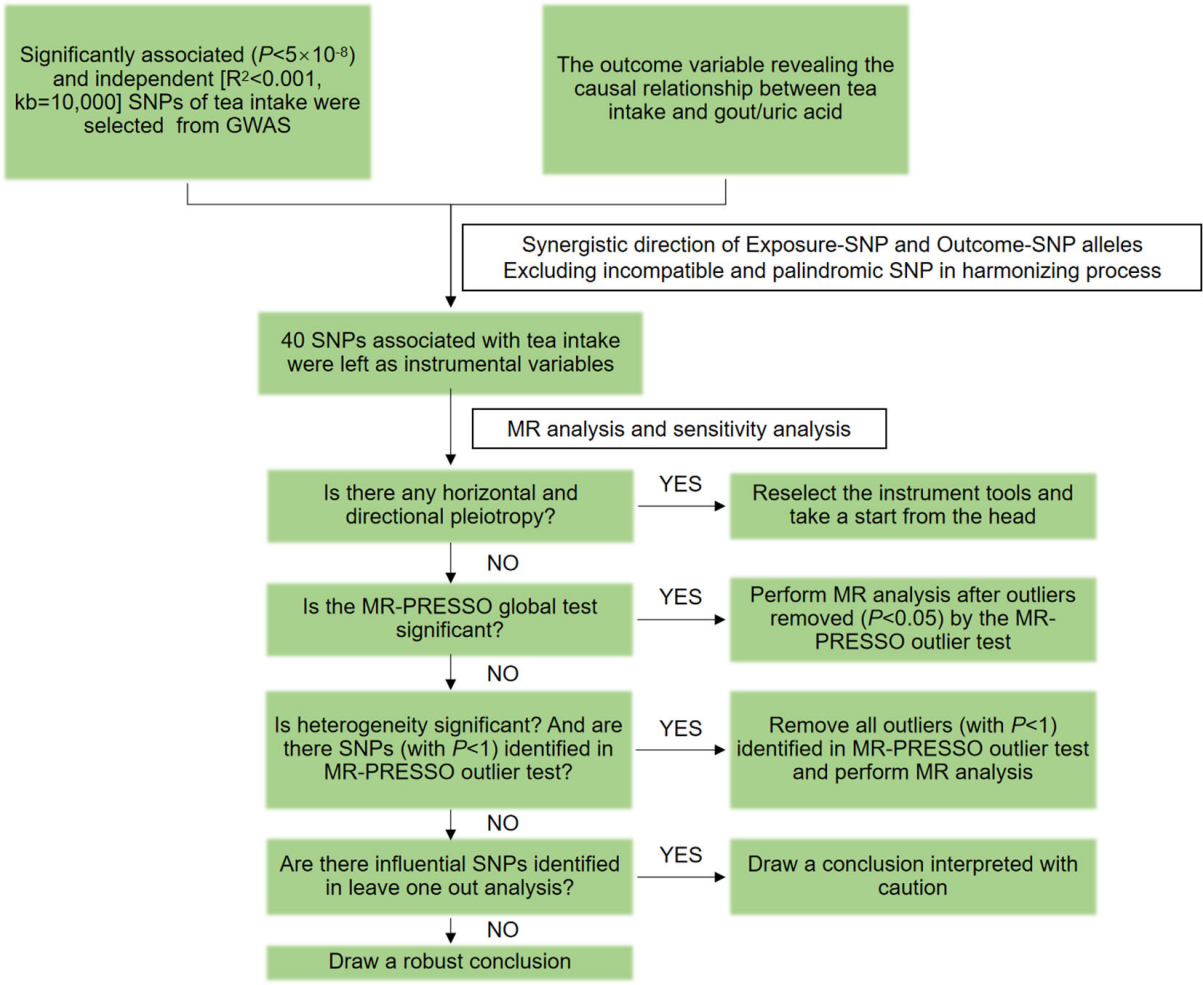
MR flow chart for causal analysis of tea intake on genetic predisposition to gout and uric acid.

This study followed the STROBE-MR guidelines (23), and the flow chart is shown in **Figure 2**. The “TwoSampleMR (0.5.7)” program package of the R 4.3.1 software was used to perform two-sample MR analysis, using inverse variance weighting (IVW), MR-Egger, and weighted median (WM) as the basic methods of assessing the causality. Among them, IVW is the primary analysis method (24), which can achieve unbiased causal estimation without horizontal pleiotropy and is the most valuable reference. MR-Egger and WM were used as supplementary tests to MR analysis. MR-Egger provides effective causal estimation in some cases of pleiotropy, and WM is less sensitive to outliers and measurement errors.

The MR-Pleiotropy Residual Sum and Outlier method (MR-PRESSO) was used to correct and analyze the MR results. After the SNPs with abnormal values (*P<* 1) were eliminated, the MR analysis was performed again. Horizontal pleiotropy was assessed using MR-Egger’s intercept analysis, with *P* ≥ 0.05 suggesting the absence of horizontal pleiotropy to meet hypothesis 3. Heterogeneity was assessed using Cochran’s Q, and *P* ≥ 0.05 indicated the absence of heterogeneity. Sensitivity analysis was performed using the leave-one-out method to assess the robustness of the MR results and to identify whether there were single SNPs that had a significant effect on the pooled results.

## 3 Results

### 3.1 GWAS data of tea intake

Data on tea intake were obtained from UK-Biobank, which included a GWAS of 447,485 Europeans. UK-biobank provided 41 SNPs strongly associated with tea intake, satisfying hypothesis 1. After reviewing the PhenoScanner V2 database and related literature, one SNP (rs1481012) associated with a known confounding variable was eliminated, satisfying hypothesis 2, as shown in **Supplementary Table S1**. While reconciling the allelic orientation of exposure-SNPs and outcome-SNPs, duplicate and mismatched SNPs were removed based on EAF value. The final included SNPs are shown in **Supplementary Table S2**.

### 3.2 GWAS data of gout and uric acid

Data on uric acid were obtained from BioBank Japan, including 109,029 East Asians, whose dataset number is bbj-a-57. Data on gout were obtained from UK Biobank, including 337,199 Europeans, whose dataset number is ukb-a-561. Gout due to impairment of renal function is derived from the FinnGen database, including 369,030 Europeans with the dataset number: finngen_R9_GOUT_KIDNEY. Data on idiopathic gout were derived from the FinnGen database, which includes 370,928 Europeans, and the dataset number was finngen_R9_GOUT_IDIO. As shown in **Table 1**.

**Table 1:** Details of the GWAS studies included in the Mendelian randomization.

Year	Trait	Population	Sample size	Web source
2018	Tea intake	European	447,485	www.nealelab.is/uk-biobank
2017	Gout	European	337,199	www.nealelab.is/uk-biobank
2023	Gout due to impairment of renal function	European	463,010	www.finngen.fi/en
2023	Idiopathic gout	European	370,928	www.finngen.fi/en
2019	Uric acid	East Asian	109,029	biobankjp.org/en/

### 3.3 MR analysis results of two samples

**Figure 3:**
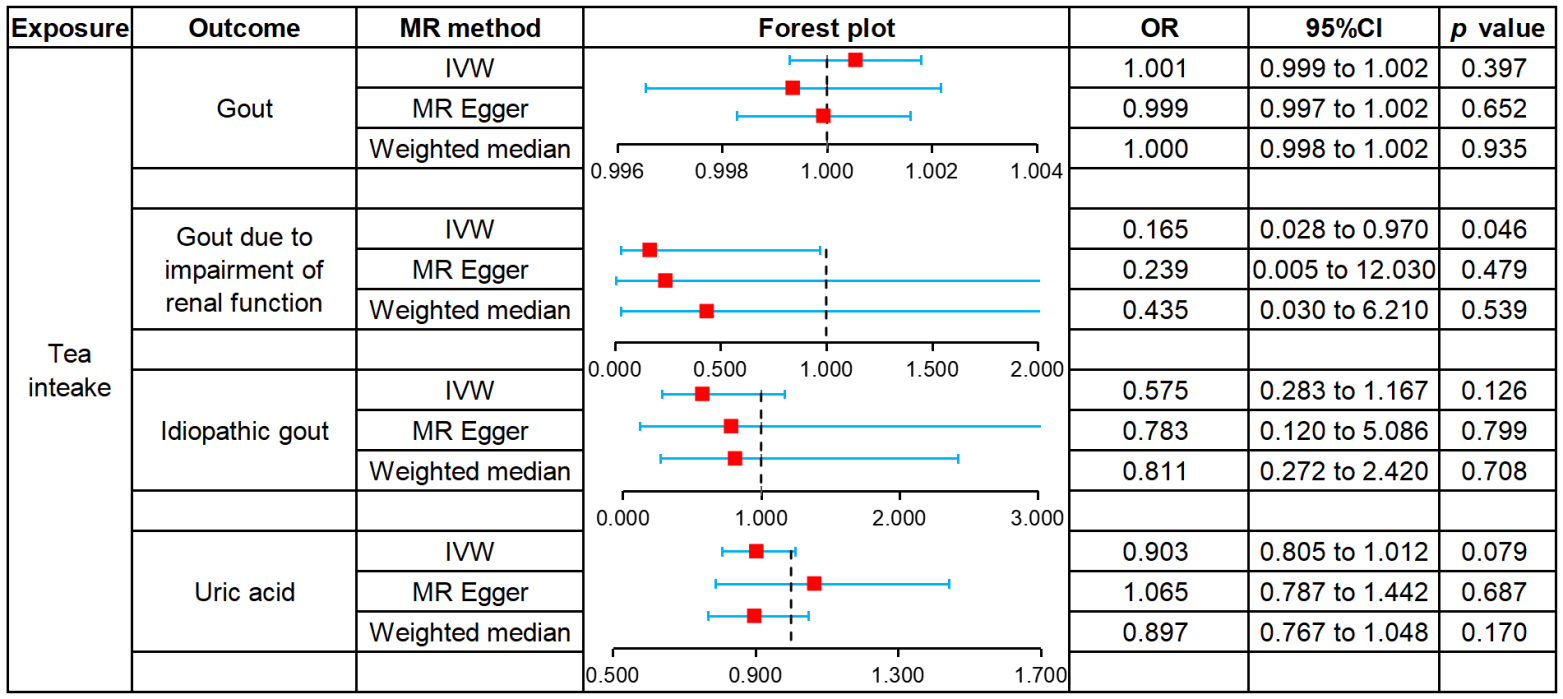
Forest plot of MR analysis for tea intake on genetic predisposition to gout and uric acid.

**Figure 4:**
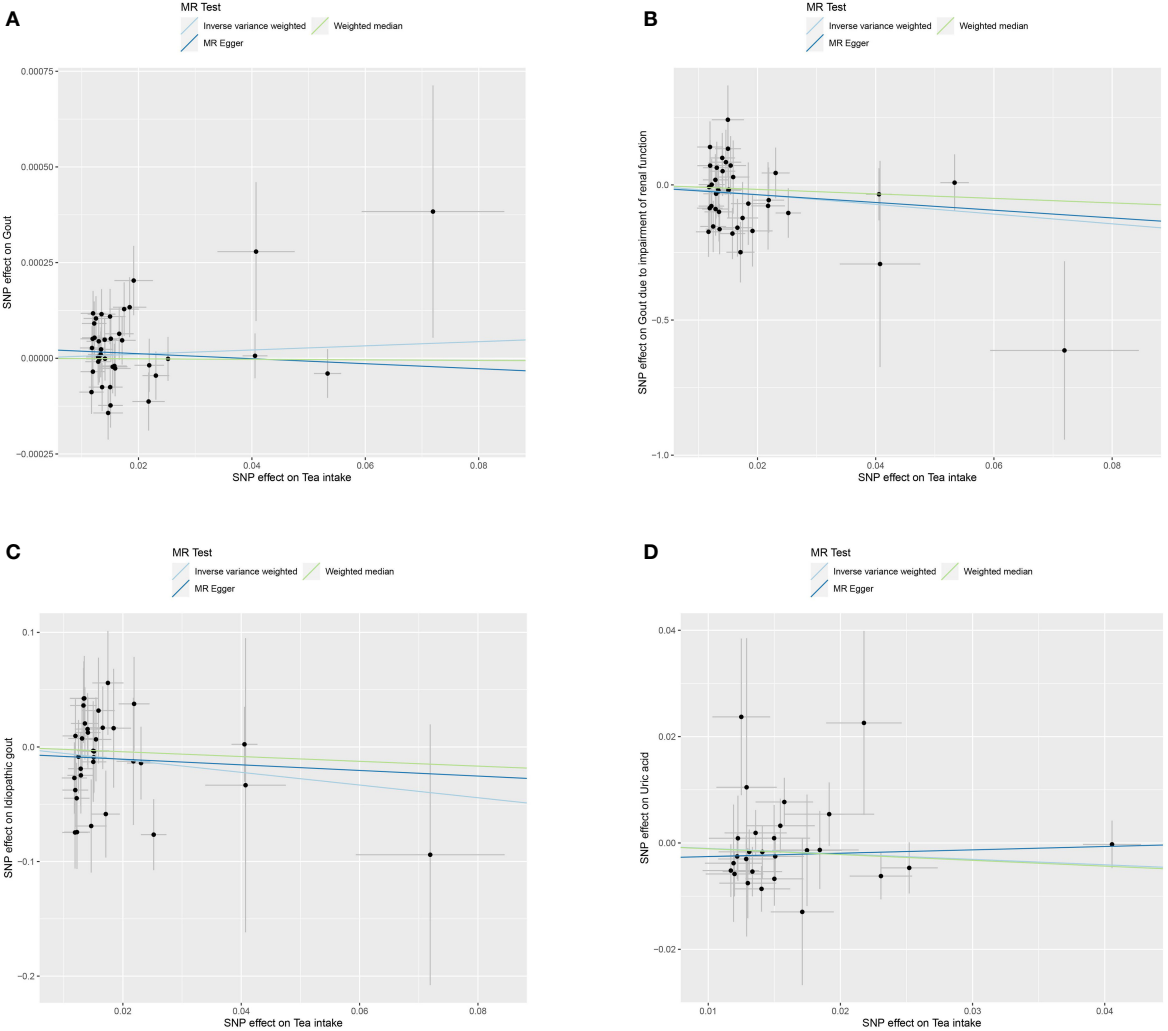
Scatter plot of MR analysis for tea intake on genetic predisposition to gout **(A)**, gout due to impairment of renal function **(B)**, idiopathic gout **(C)**, uric acid **(D)**.

We used MR to analyze the effect of tea intake on genetic predisposition to gout, gout due to impairment of renal function, idiopathic gout as well as uric acid. The forest plot of the MR analysis is shown in **Figure 3**, and the effect estimates for each SNP are indicated in **Figure 4**. The intercept analysis of MR-Egger is demonstrated in **Supplementary Table S3**, the heterogeneity test results are displayed in **Supplementary Figure S1** and **Supplementary Table S4**, and the sensitivity analysis results is shown in **Supplementary Figure S2**.

#### 3.3.1 Gout

All three methods of analysis showed no significant effect of tea intake on genetic predisposition to gout: IVW (OR 1.001, 95% CI 0.999 to 1.002, *P* = 0.397), MR-Egger (OR 0.999, 95% CI 0.997 to 1.002, *P* = 0.652), WM (OR 1.000, 95% CI 0.998 to 1.002, *P* = 0.935). Intercept analysis showed no horizontal pleiotropy (*P* = 0.359). The heterogeneity test revealed the absence of heterogeneity (*P* = 0.056). Leave-one-out sensitivity analysis verified the stability of the results.

#### 3.3.2 Gout due to impairment of renal function

IVW analysis showed that increased tea intake was associated with reduced genetic susceptibility to gout due to impairment of renal function (OR 1.001, 95% CI 0.999 to 1.002, *P* = 0.397), which was not observed in MR-Egger (OR 0.239, 95% CI 0.005 to 12.030, *P* = 0.479) and WM (OR 0.435, 95% CI 0.030 to 6.210, *P* = 0.539). Intercept analysis indicated there was no horizontal pleiotropy (*P* = 0.837). The heterogeneity test showed no heterogeneity (*P* = 0.414). Leave-one-out sensitivity analysis verified the stability of the results.

#### 3.3.3 Idiopathic gout

All three methods of analysis showed no significant effect of tea intake on genetic predisposition to idiopathic gout: IVW(OR 0.575, 95% CI 0.283 to 1.167, *P* = 0.126), MR-Egger (OR 0.783, 95% CI 0.120 to 5.086, *P* = 0.799), WM (OR 0.811, 95% CI 0.272 to 2.420, *P* = 0.708). Intercept analysis showed no horizontal pleiotropy (*P* = 0.728). The heterogeneity test revealed the absence of heterogeneity (*P* = 0.388). Leave-one-out sensitivity analysis verified the stability of the results.

#### 3.3.4 Uric acid

All three methods of analysis showed no significant effect of tea intake on genetic predisposition to uric acid: IVW (OR 0.903, 95% CI 0.805 to 1.012, *P* = 0.079), MR-Egger (OR 1.065, 95% CI 0.787 to 1.442, *P* = 0.687), WM(OR 897, 95% CI 0.767 to 1.048, *P* = 0.170). Intercept analysis revealed no horizontal pleiotropy (*P* = 0.258). The heterogeneity test showed no heterogeneity (*P* = 0.654). Leave-one-out sensitivity analysis verified the stability of the results.

## 4 Discussion

Gout is a chronic disease caused by the deposition of urate crystals in and/or around the joints (25), and its onset is mainly related to excessive secretion and/or insufficient renal excretion of uric acid (26). Uric acid is the end product of purine nucleotide metabolism in the body (27), with approximately two-thirds of purines being endogenous and the remaining one-third being exogenous purines that enter the body through food (28). After the dephosphorylation and oxidation of exogenous purines, the degraded bases can be degraded into uric acid in the liver or small intestine (29). Approximately 90% of uric acid in the body is reabsorbed, and the remainder is excreted in urine or feces (30). When the purine content of the body exceeds the threshold for production and catabolism, the balance between uric acid synthesis and excretion is disrupted, resulting in a constant rise in blood uric acid and a significant increase in the risk of gout (31). At present, obesity, alcohol consumption, and a high-purine diet are considered to be risk factors for the onset of gout (32–34), while the role of tea drinking in regulating uric acid and affecting the onset of gout is still controversial. Tea is one of the most popular beverages worldwide and contains various bioactive compounds such as polyphenols, theaflavins, free amino acids, and purine alkaloids (35). Studies have shown that tea can reduce uric acid, improve kidney function, and reduce body weight (36–38). However, it has also been reported that long-term tea drinking has no significant effect on uric acid (39) and can even increase uric acid (40). In order to find out the correlation between tea drinking and gout as well as uric acid, this study used MR to explore the effect of tea intake on genetic predisposition to gout, gout due to impairment of renal function, idiopathic gout and uric acid.

The results of this study demonstrated that tea intake has no significant genetic predisposition effect on uric acid, gout, or idiopathic gout, whereas there was a significant effect on the genetic predisposition of gout due to impairment of renal function. The heterogeneity test showed no heterogeneity in this study’s results; the leave-one-out sensitivity analysis also proved the stability of the results, and no horizontal polytropy was observed in the intercept analysis. It is worth noting that the data related to tea intake, gout, gout due to impairment of renal function, and idiopathic gout in this study are from Europeans, while the data related to uric acid are from East Asians. This is because the GWAS only provides uric acid for East Asians, and racial differences may lead to less confidence in the result of uric acid. The types of tea in Europe and East Asia are different, and the ethnic composition is also different, so both the type of tea and the ethnic group may be potential factors affecting the analysis results. Furthermore, tea intake can reduce the risk of gout due to impairment of renal function without a significant effect on uric acid. This may be related to the different sources of data, and the data collected in different study designs may have inconsistent effects on the results. Therefore, further studies are needed to elucidate the effect and mechanism of tea intake on gout and uric acid.

In previous cohort studies, the relationship between tea intake and gout as well as uric acid has been inconsistent. In an earlier study, Conlay LA et al. (41) found that a dosage of 65 ± 10 mg/(kg·d) of caffeine, the active ingredient in tea, elevated plasma adenosine levels in a dose-related and saturable manner. Considering that adenosine is involved in uric acid synthesis, they hypothesized that tea might increase uric acid levels. However, in a regression analysis, Liu A et al. (42) reported a nonlinear and negative association between caffeine intake and serum uric acid levels in men, while no such association was observed in women. It is evident that the impact of caffeine on serum uric acid levels remains a subject of debate. More studies support the role of other active tea ingredients in lowering uric acid. Chen G et al. (43) found that green tea and green tea polyphenols could reduce uric acid levels in rats by decreasing uric acid production and increasing uric acid excretion. Shi Y et al. (44) showed that quercetin, a flavonoid in high amounts in black tea, could reduce plasma uric acid levels in healthy patients. A animal experiment by Yuan D et al. (45) showed that black brick tea with a high theaflavin content had a better blood uric acid-lowering effect than black tea. In a 3-week clinical study, Jatuworapruk K et al. (46) found that catechins, an extract of tea, reduced uric acid levels and increased serum antioxidant capacity in healthy individuals in the short term. Subsequent studies have shown that the uric acid-lowering effect of catechins stems from the fact that it promotes the excretion of uric acid as well as uric acid precursors (47). Interestingly, a subgroup analysis by Peluso I et al. (48) showed that the tea extract teaflavanol significantly lowered the uric acid levels of patients with gout but significantly elevated the uric acid levels of normal subjects, with a mechanism of action that may be related to the regulation of xanthine oxidase. In summary, caffeine, the active ingredient of tea, has a controversial effect on uric acid, and other active ingredients such as tea polyphenols, quercetin, theaflavins, flavanols, and catechins have the effect of decreasing uric acid, whereas theaflavanols demonstrate a bidirectional modulating effect. These pieces of evidence reflect the different effects of different active ingredients of tea on uric acid, but they do not explain the effect of tea as a whole on uric acid.

Although caffeine, the active ingredient in tea, has been suggested to cause an increase in serum uric acid levels potentially, there is no relevant clinical evidence to support this contention. Clinical studies to date suggest that tea may or may not be associated with reduced serum uric acid levels. For one thing, Zhang ZX et al. (49) found that tea lowered serum uric acid levels by inhibiting uric acid production. Wu D et al. (35) showed that green, yellow, white, oolong, black, and black teas significantly inhibited xanthine oxidase, which, in turn, exerted a uric acid-lowering effect. Yuan D et al. (45) reported that black tea reduced uric acid levels both by inhibiting hepatic xanthine oxidase (XOD) and adenosine deaminase (ADA) activity to reduce uric acid production and also to increase uric acid excretion by modulating renal urate transporter protein expression. These studies suggest that tea may reduce serum uric acid levels by inhibiting uric acid production and promoting uric acid excretion. For another, Chen DD et al. (49) found no significant correlation between tea intake and uric acid levels in Chinese. In a study based on the Third National Health and Nutrition Examination Survey in the United States, Choi HK et al. (39) found that tea consumption did not reduce uric acid or the incidence of hyperuricemia. A Korean cohort study also showed that increased tea intake did not affect the risk of hyperuricemia (50). Although the results of clinical studies are controversial, published meta-analyze have confirmed that tea is not associated with serum uric acid levels or the risk of gout (51), which supports our findings. In this MR analysis, we did not find an effect of tea intake on genetic predisposition to uric acid, gout, or idiopathic gout.

Of note, this study also found that increased tea intake was associated with a reduced risk of gout due to impairment of renal function. This effect may be related to its role in protecting renal function and regulating intestinal function. First, the effect of tea in reducing the risk of gout due to impairment of renal function may be realized by improving renal function. Uric acid excretion mainly involves reabsorption through glomerular filtration, renal tubules, and collecting ducts (52). Tea has been reported to modulate the expression of organic anion transporter protein 1 (OAT1) and organic anion transporter protein 3 (OAT3) and to downregulate the expression of uric acid transporter protein 1 (URAT1) and glucose transporter protein 9 (GLUT9) in the kidney, in order to increase renal excretion of uric acid and decrease uric acid reabsorption (53, 54). It has also been shown that tea reduces the formation of inflammatory factors induced by urate crystals and inhibits the activation of the NLRP3 inflammatory vesicle and NF-κB pathway (55), thereby decreasing the likelihood of gout in patients (45). Zhang Y et al. (56) found that tea increases the eGFR level and reduces the risk of chronic kidney disease and proteinuria. Ding Z et al. (57) reported that tea protects renal tubular epithelial cells by inhibiting the pyroptosis pathway, thereby attenuating the damage caused by uric acid to the kidneys. It has also been reported that tea and its extracts inhibit the growth of glomerular membrane cells, thereby delaying the progression of renal failure (58, 59). In a prospective cohort study of 447,658 individuals, Guo H et al. (60) found that drinking more than 6 cups of tea per day decreased the risk of gout by 23%, suggesting a clear link between tea and gout reduction. Intriguingly, the effect of tea in reducing the risk of gout in our study occurred only in gout due to impairment of renal function and not in all types of gout, which may be because the renoprotective effect of tea was more pronounced in patients with pre-existing renal impairment.

Second, the role of tea in reducing the risk of gout due to impairment of renal function may also be related to the regulation of intestinal function. In addition to renal excretion, total uric acid in the human body can be excreted through the liver and biliary system to the intestinal tract (61). The intestinal tract can be the main route of uric acid excretion when the human kidneys fail (62). ABCG2 is a primary transporter protein for uric acid excretion in the intestine (63). Studies have shown that tea-active ingredients can promote uric acid excretion in the intestine (52) and reduce the metabolism of purines and pyrimidines by up-regulating the expression of intestinal ABCG2 (64, 65). In recent years, with the in-depth research related to intestinal flora, some scholars have realized that intestinal flora is expected to be a new target for treating hyperuricemia (66). The active ingredients of tea have been reported to modulate the composition and number of intestinal flora and to reduce intestinal dysbiosis induced by a high-fat diet (53). In addition, tea can reduce the number of bile salt hydrolase-containing bacteria in the intestinal tract as well as the functional activity of bile salt hydrolase, thus exerting a weight loss effect (14, 67). And obesity is one of the risk factors for the development of gout (32). In summary, the role of tea in reducing the risk of gout due to impairment of renal function may also be related to modulation of intestinal function, as intestinal excretion of uric acid is more important in patients with renal impairment or failure.

This study analyzed the causal association of tea with gout and uric acid using genetic variants provided by GWAS. The researchers observed that the genetic predisposition of gout due to impairment of renal function decreased with increasing tea intake. This finding can help guide the use of tea in patients with renal insufficiency. Firstly, clinicians can advise patients with renal insufficiency to drink tea in moderation to reduce the risk of gout. Secondly, patients with pre-existing gout due to impairment of renal function may be able to slow progression and improve prognosis by drinking tea. By elucidating these links, tea can be recommended as part of a holistic approach to the management of gout due to impairment of renal function, thereby optimizing patient dietary care.

This study also has some limitations. First, the results of this study are based on GWAS data, and we did not conduct a large-scale cohort study or genomic validation due to funding constraints. Second, because the study data were derived from self-reported data of GWAS, we could not obtain accurate tea intake and entirely exclude interference from other dietary and lifestyle factors, which could have reduced the precision of the results. Third, due to the wide variety of teas, the results of this study cannot be used to explain the effects of different types of tea on gout and uric acid. Fourth, the study included European and East Asian populations and did not include Africans and Latinos, which may have reduced the generalizability of the results. Fifth, Although the sensitivity analysis results were robust, other factors’ effects on gout and uric acid could not be entirely excluded.

Given the above limitations, we look forward to continued improvements in future studies. First, research centers can be set up in different continents and countries, and then large-scale cohort studies of tea and uric acid as well as tea and gout can be promoted, thereby providing more and more comprehensive data for MR. Second, stratified research can be carried out by controlling related variables to explore the effects of different types of tea on healthy people, hyperuricemia patients, and different types of gout patients.

## 5 Conclusion

This MR analysis suggested that increased tea intake was associated with reduced genetic predisposition to gout due to impairment of renal function, independent of gout, idiopathic gout, and uric acid. And tea intake may become an essential program in the dietary treatment of gout due to impairment of renal function. In the future, more research is needed to explore the effect and mechanism of tea intake on gout and uric acid.

## Data availability statement

The original contributions presented in the study are included in the article/**Supplementary Material**. Further inquiries can be directed to the corresponding author.

## Author contributions

YYu: Conceptualization, Data curation, Supervision, Writing – original draft. XY: Methodology, Supervision, Writing – original draft. GH: Data curation, Methodology, Writing – original draft. KT: Data curation, Formal analysis, Writing – original draft. YYi: Formal analysis, Methodology, Writing – original draft. RY: Writing – review & editing.
