# Supplementary figures and images for: Effect of tea intake on genetic predisposition to gout and uric acid: a Mendelian randomization study
Source: Front Endocrinol (Lausanne). 2024 Feb 2;14:1290731. doi: 10.3389/fendo.2023.1290731 (PMC10911082; doi:10.3389/fendo.2023.1290731)

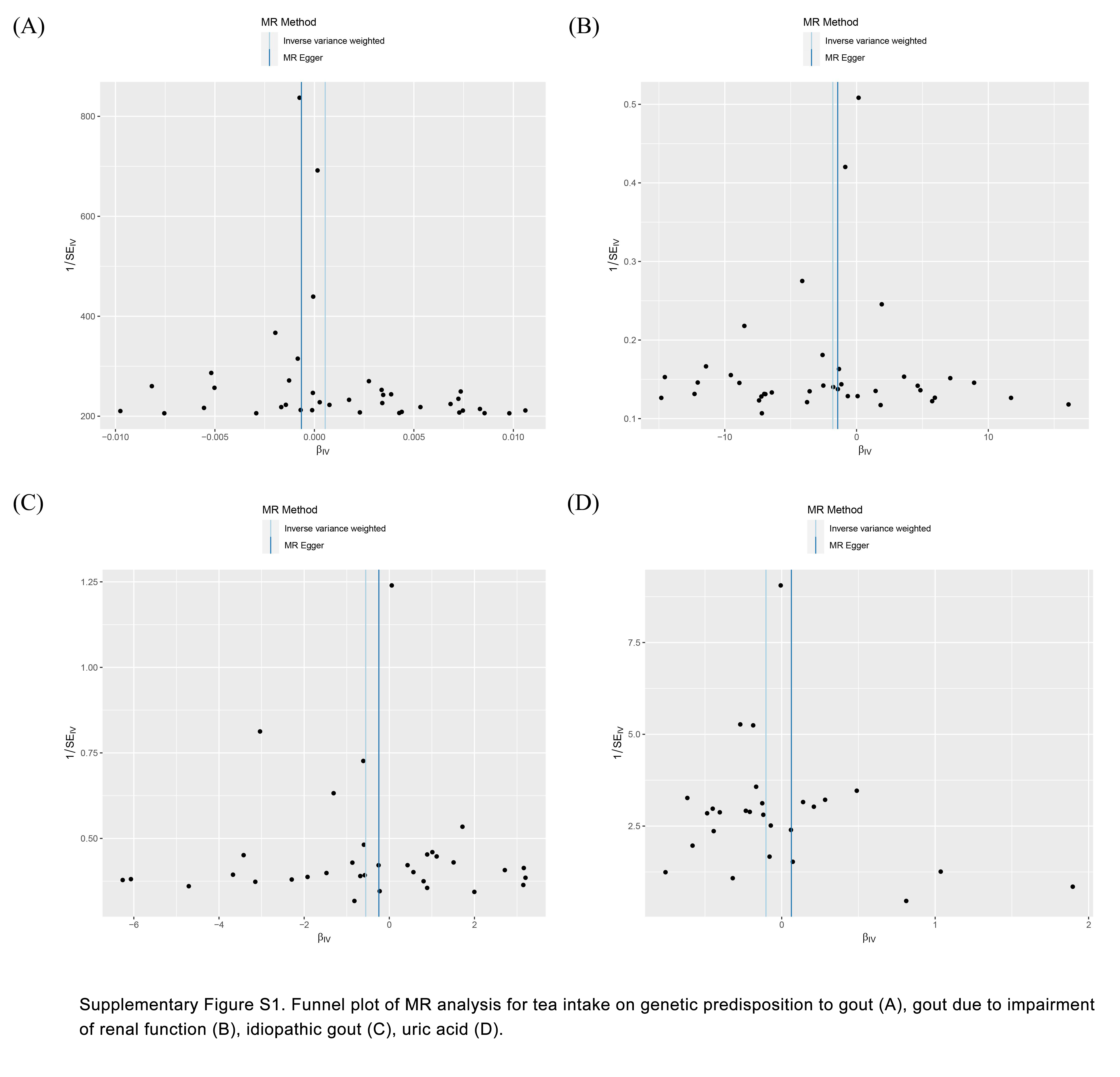

Supplement: Supplementary file 1 [file Image_1.jpg]

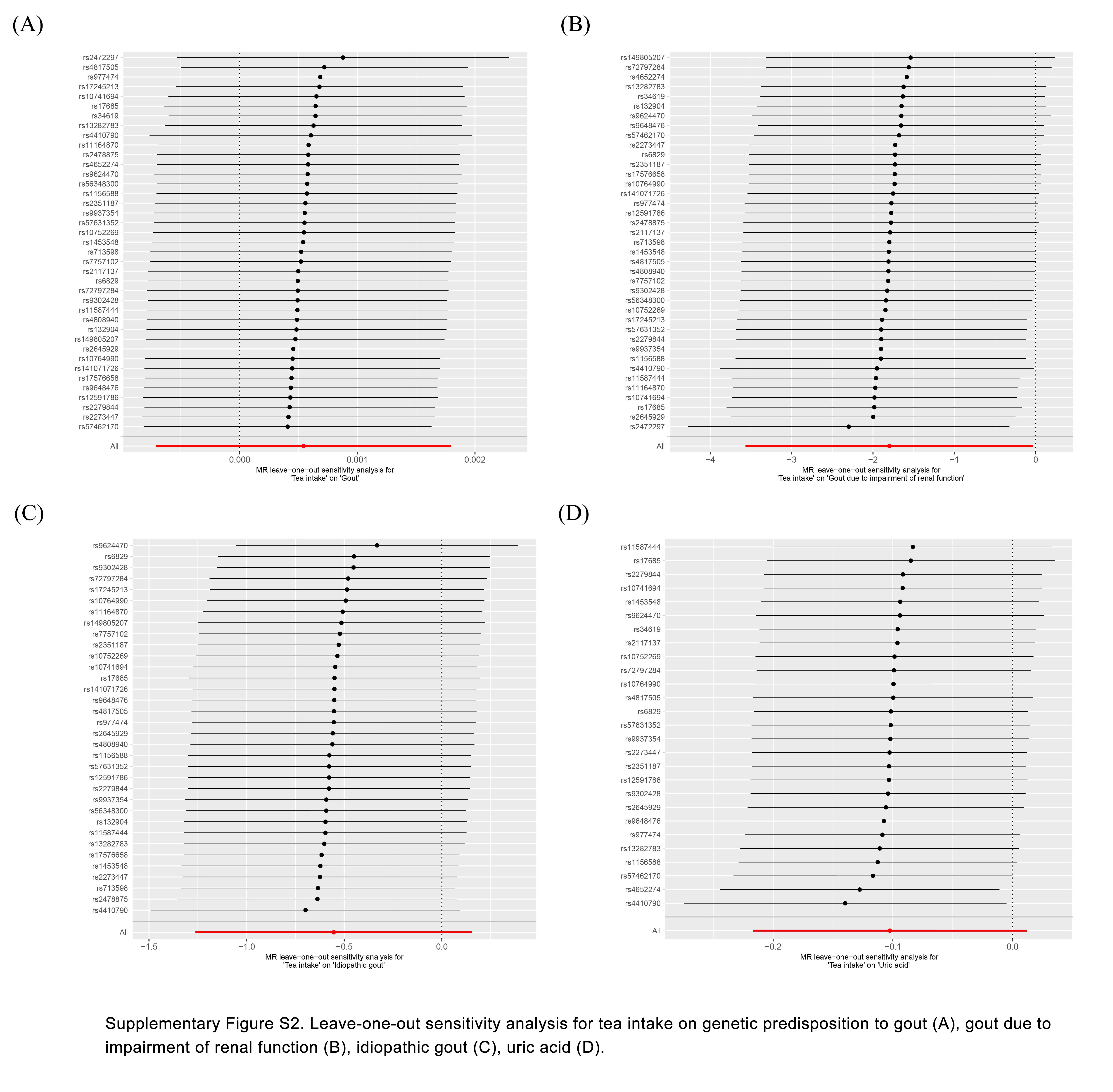

Supplement: Supplementary file 2 [file Image_2.jpg]
