# Supplementary material for: Effect of tea intake on genetic predisposition to gout and uric acid: a Mendelian randomization study
Source: Front Endocrinol (Lausanne). 2024 Feb 2;14:1290731. doi: 10.3389/fendo.2023.1290731 (PMC10911082; doi:10.3389/fendo.2023.1290731)
Supplement: Supplementary file 3 [file Table_1.docx]

**Supplementary Table S1** Characteristics of instrumental variables for tea intake

|  | **SNP** | **EA** | **OA** | **Samplesize** | **SE** | **β** | **id.exposure** | **EAF** | ***P* value** | **R^2^** | **F - statistic** |
| --- | --- | --- | --- | --- | --- | --- | --- | --- | --- | --- | --- |
| 1 | rs11164870 | G | C | 447485 | 0.00218232 | -0.0119604 | ukb-b-6066 | 0.604574 | 0.604574 | 6.84E-05 | 30.60852054 |
| 2 | rs11587444 | G | A | 447485 | 0.00217078 | 0.0140328 | ukb-b-6066 | 0.393464 | 0.393464 | 9.40E-05 | 42.06274532 |
| 3 | rs4652274 | C | T | 447485 | 0.0021744 | -0.0157568 | ukb-b-6066 | 0.387218 | 0.387218 | 0.000117822 | 52.72969893 |
| 4 | rs1156588 | G | A | 447485 | 0.00260325 | -0.015454 | ukb-b-6066 | 0.210071 | 0.210071 | 7.93E-05 | 35.47128631 |
| 5 | rs57462170 | A | G | 447485 | 0.00340563 | 0.0191505 | ukb-b-6066 | 0.108773 | 0.108773 | 7.11E-05 | 31.82050699 |
| 6 | rs2117137 | G | A | 447485 | 0.0021557 | 0.0129948 | ukb-b-6066 | 0.405148 | 0.405148 | 8.14E-05 | 36.4253482 |
| 7 | rs34619 | A | G | 447485 | 0.0021375 | 0.0117117 | ukb-b-6066 | 0.430905 | 0.430905 | 6.73E-05 | 30.10522903 |
| 8 | rs72797284 | G | A | 447485 | 0.00238353 | -0.0171147 | ukb-b-6066 | 0.270797 | 0.270797 | 0.000115681 | 51.77113142 |
| 9 | rs2478875 | G | A | 447485 | 0.00261129 | 0.0218943 | ukb-b-6066 | 0.208758 | 0.208758 | 0.00015836 | 70.87448326 |
| 10 | rs149805207 | G | A | 447485 | 0.0125823 | -0.0719337 | ukb-b-6066 | 0.008538 | 0.008538 | 8.76E-05 | 39.20501346 |
| 11 | rs7757102 | G | A | 447485 | 0.00213302 | -0.0118039 | ukb-b-6066 | 0.555426 | 0.555426 | 6.88E-05 | 30.79340618 |
| 12 | rs4410790 | C | T | 447485 | 0.00219507 | 0.0405506 | ukb-b-6066 | 0.631224 | 0.631224 | 0.000765545 | 342.83082 |
| 13 | rs141071726 | A | G | 447485 | 0.00681204 | 0.0407321 | ukb-b-6066 | 0.026713 | 0.026713 | 8.63E-05 | 38.60834585 |
| 14 | rs9648476 | A | G | 447485 | 0.00218542 | 0.0125013 | ukb-b-6066 | 0.622954 | 0.622954 | 7.34E-05 | 32.85482007 |
| 15 | rs17685 | A | G | 447485 | 0.00236195 | 0.0230655 | ukb-b-6066 | 0.277512 | 0.277512 | 0.000213338 | 95.48548326 |
| 16 | rs713598 | G | C | 447485 | 0.00215659 | 0.0133969 | ukb-b-6066 | 0.402254 | 0.402254 | 8.63E-05 | 38.62510767 |
| 17 | rs13282783 | T | C | 447485 | 0.00235432 | -0.0135837 | ukb-b-6066 | 0.285899 | 0.285899 | 7.53E-05 | 33.71690476 |
| 18 | rs56348300 | G | C | 447485 | 0.00273191 | 0.0158824 | ukb-b-6066 | 0.184619 | 0.184619 | 7.59E-05 | 33.98668146 |
| 19 | rs10752269 | A | G | 447485 | 0.00211975 | -0.0128727 | ukb-b-6066 | 0.506082 | 0.506082 | 8.28E-05 | 37.07298506 |
| 20 | rs2351187 | A | G | 447485 | 0.0022823 | 0.0129023 | ukb-b-6066 | 0.318935 | 0.318935 | 7.23E-05 | 32.36405728 |
| 21 | rs10764990 | A | G | 447485 | 0.00216898 | -0.0121906 | ukb-b-6066 | 0.607155 | 0.607155 | 7.09E-05 | 31.72548679 |
| 22 | rs17245213 | A | G | 447485 | 0.00260905 | -0.0146481 | ukb-b-6066 | 0.208046 | 0.208046 | 7.07E-05 | 31.64165303 |
| 23 | rs10741694 | C | T | 447485 | 0.00219355 | 0.0150037 | ukb-b-6066 | 0.627915 | 0.627915 | 0.000105189 | 47.07517964 |
| 24 | rs1453548 | A | T | 447485 | 0.00224973 | -0.0133414 | ukb-b-6066 | 0.664929 | 0.664929 | 7.93E-05 | 35.49407889 |
| 25 | rs977474 | T | C | 447485 | 0.00285559 | 0.0217813 | ukb-b-6066 | 0.833746 | 0.833746 | 0.000131524 | 58.86228541 |
| 26 | rs2645929 | G | A | 447485 | 0.0027166 | -0.0149842 | ukb-b-6066 | 0.813066 | 0.813066 | 6.83E-05 | 30.54340147 |
| 27 | rs2783129 | G | C | 447485 | 0.00213314 | -0.0117331 | ukb-b-6066 | 0.484878 | 0.484878 | 6.88E-05 | 30.7754581 |
| 28 | rs17576658 | A | G | 447485 | 0.00245655 | -0.0134812 | ukb-b-6066 | 0.247081 | 0.247081 | 6.76E-05 | 30.26081463 |
| 29 | rs6829 | T | C | 447485 | 0.00216546 | -0.0119163 | ukb-b-6066 | 0.596155 | 0.596155 | 6.84E-05 | 30.59799642 |
| 30 | rs12591786 | T | C | 447485 | 0.00294243 | -0.0184399 | ukb-b-6066 | 0.158804 | 0.158804 | 9.08E-05 | 40.65572889 |
| 31 | rs2472297 | T | C | 447485 | 0.00240098 | 0.0533453 | ukb-b-6066 | 0.262049 | 0.262049 | 0.001100607 | 493.0456637 |
| 32 | rs9302428 | G | C | 447485 | 0.00220122 | 0.0122457 | ukb-b-6066 | 0.635799 | 0.635799 | 6.94E-05 | 31.07884742 |
| 33 | rs9937354 | A | G | 447485 | 0.0021433 | -0.0140923 | ukb-b-6066 | 0.424074 | 0.424074 | 9.70E-05 | 43.41309613 |
| 34 | rs2279844 | A | G | 447485 | 0.00218318 | -0.0119879 | ukb-b-6066 | 0.379343 | 0.379343 | 6.77E-05 | 30.28348709 |
| 35 | rs57631352 | G | A | 447485 | 0.00232117 | -0.0131035 | ukb-b-6066 | 0.296859 | 0.296859 | 7.17E-05 | 32.07782668 |
| 36 | rs4808940 | G | C | 447485 | 0.00224718 | 0.015123 | ukb-b-6066 | 0.335314 | 0.335314 | 0.000101947 | 45.62416573 |
| 37 | rs2273447 | T | A | 447485 | 0.00263421 | 0.0174715 | ukb-b-6066 | 0.203788 | 0.203788 | 9.91E-05 | 44.33199452 |
| 38 | rs4817505 | C | T | 447485 | 0.0021746 | 0.015068 | ukb-b-6066 | 0.38998 | 0.38998 | 0.000108026 | 48.34494675 |
| 39 | rs9624470 | A | G | 447485 | 0.00215485 | 0.0252071 | ukb-b-6066 | 0.580054 | 0.580054 | 0.000309555 | 138.5634344 |
| 40 | rs132904 | C | G | 447485 | 0.00255257 | 0.0166007 | ukb-b-6066 | 0.778651 | 0.778651 | 9.50E-05 | 42.51292702 |

SNP, single nucleotide polymorphism; EA, effect allele; OA, other allele; EAF, effect allele frequency; SE, standard error.

**Supplementary Table S2.1** SNPs from GWAS on tea intake and gout

|  | | | **Exposure (Tea intake)** | | |  | **Outcome (Gout)** | | | | |
| --- | --- | --- | --- | --- | --- | --- | --- | --- | --- | --- | --- |
| **SNP** | **EA** | **OA** | **β** | **SE** | ***P* value** |  | **Case** | **Control** | **β** | **SE** | ***P* value** |
| rs10741694 | C | T | 0.0150037 | 0.00219355 | 7.92E-12 |  | 181 | 337,018 | -7.54E-05 | 5.84E-05 | 0.197078841 |
| rs10752269 | A | G | -0.0128727 | 0.00211975 | 1.26E-09 |  | 181 | 337,018 | -3.44E-06 | 5.65E-05 | 0.95149191 |
| rs10764990 | A | G | -0.0121906 | 0.00216898 | 1.90E-08 |  | 181 | 337,018 | -9.10E-05 | 5.77E-05 | 0.114888088 |
| rs11164870 | G | C | -0.0119604 | 0.00218232 | 4.24E-08 |  | 181 | 337,018 | 3.50E-05 | 5.81E-05 | 0.547202408 |
| rs1156588 | G | A | -0.015454 | 0.00260325 | 2.91E-09 |  | 181 | 337,018 | 2.22E-05 | 6.94E-05 | 0.749152098 |
| rs11587444 | G | A | 0.0140328 | 0.00217078 | 1.02E-10 |  | 181 | 337,018 | 4.85E-05 | 5.78E-05 | 0.401604897 |
| rs12591786 | T | C | -0.0184399 | 0.00294243 | 3.68E-10 |  | 181 | 337,018 | -0.000133459 | 7.85E-05 | 0.089258585 |
| rs13282783 | T | C | -0.0135837 | 0.00235432 | 7.94E-09 |  | 181 | 337,018 | 7.54E-05 | 6.27E-05 | 0.229420911 |
| rs132904 | C | G | 0.0166007 | 0.00255257 | 7.85E-11 |  | 181 | 337,018 | 6.40E-05 | 6.81E-05 | 0.347065145 |
| rs141071726 | A | G | 0.0407321 | 0.00681204 | 2.24E-09 |  | 181 | 337,018 | 0.000278715 | 0.000181515 | 0.124663983 |
| rs1453548 | A | T | -0.0133414 | 0.00224973 | 3.03E-09 |  | 181 | 337,018 | -1.01E-05 | 5.99E-05 | 0.866261075 |
| rs149805207 | G | A | -0.0719337 | 0.0125823 | 1.08E-08 |  | 181 | 337,018 | -0.000383341 | 0.000329586 | 0.244790185 |
| rs17245213 | A | G | -0.0146481 | 0.00260905 | 1.97E-08 |  | 181 | 337,018 | 0.000142874 | 6.97E-05 | 0.040249455 |
| rs17576658 | A | G | -0.0134812 | 0.00245655 | 4.07E-08 |  | 181 | 337,018 | -0.000115304 | 6.54E-05 | 0.078004562 |
| rs17685 | A | G | 0.0230655 | 0.00236195 | 1.58E-22 |  | 181 | 337,018 | -4.53E-05 | 6.29E-05 | 0.47128216 |
| rs2117137 | G | A | 0.0129948 | 0.0021557 | 1.66E-09 |  | 181 | 337,018 | 4.43E-05 | 5.74E-05 | 0.440107734 |
| rs2273447 | T | A | 0.0174715 | 0.00263421 | 3.30E-11 |  | 181 | 337,018 | 0.000128498 | 7.00E-05 | 0.06657482 |
| rs2279844 | A | G | -0.0119879 | 0.00218318 | 4.00E-08 |  | 181 | 337,018 | -0.000117447 | 5.82E-05 | 0.043747174 |
| rs2351187 | A | G | 0.0129023 | 0.0022823 | 1.57E-08 |  | 181 | 337,018 | -8.92E-06 | 6.08E-05 | 0.883278989 |
| rs2472297 | T | C | 0.0533453 | 0.00240098 | 2.29E-109 |  | 181 | 337,018 | -3.98E-05 | 6.37E-05 | 0.532121737 |
| rs2478875 | G | A | 0.0218943 | 0.00261129 | 5.10E-17 |  | 181 | 337,018 | -1.83E-05 | 6.95E-05 | 0.791769923 |
| rs2645929 | G | A | -0.0149842 | 0.0027166 | 3.47E-08 |  | 181 | 337,018 | -0.000109139 | 7.23E-05 | 0.131055122 |
| rs34619 | A | G | 0.0117117 | 0.0021375 | 4.27E-08 |  | 181 | 337,018 | -8.84E-05 | 5.69E-05 | 0.120453105 |
| rs4410790 | C | T | 0.0405506 | 0.00219507 | 3.38E-76 |  | 181 | 337,018 | 6.39E-06 | 5.86E-05 | 0.913162987 |
| rs4652274 | C | T | -0.0157568 | 0.0021744 | 4.28E-13 |  | 181 | 337,018 | 2.00E-05 | 5.80E-05 | 0.729788474 |
| rs4808940 | G | C | 0.015123 | 0.00224718 | 1.70E-11 |  | 181 | 337,018 | 5.11E-05 | 5.99E-05 | 0.393415077 |
| rs4817505 | C | T | 0.015068 | 0.0021746 | 4.24E-12 |  | 181 | 337,018 | -0.000123129 | 5.79E-05 | 0.033542081 |
| rs56348300 | G | C | 0.0158824 | 0.00273191 | 6.11E-09 |  | 181 | 337,018 | -2.65E-05 | 7.28E-05 | 0.716039532 |
| rs57462170 | A | G | 0.0191505 | 0.00340563 | 1.87E-08 |  | 181 | 337,018 | 0.00020308 | 9.06E-05 | 0.024919539 |
| rs57631352 | G | A | -0.0131035 | 0.00232117 | 1.65E-08 |  | 181 | 337,018 | 1.49E-06 | 6.18E-05 | 0.980815996 |
| rs6829 | T | C | -0.0119163 | 0.00216546 | 3.74E-08 |  | 181 | 337,018 | -5.08E-05 | 5.77E-05 | 0.378588136 |
| rs713598 | G | C | 0.0133969 | 0.00215659 | 5.23E-10 |  | 181 | 337,018 | 2.33E-05 | 5.76E-05 | 0.685215219 |
| rs72797284 | G | A | -0.0171147 | 0.00238353 | 6.95E-13 |  | 181 | 337,018 | -4.69E-05 | 6.34E-05 | 0.459528022 |
| rs7757102 | G | A | -0.0118039 | 0.00213302 | 3.13E-08 |  | 181 | 337,018 | -2.70E-05 | 5.69E-05 | 0.635249015 |
| rs9302428 | G | C | 0.0122457 | 0.00220122 | 2.65E-08 |  | 181 | 337,018 | 5.38E-05 | 5.87E-05 | 0.359876096 |
| rs9624470 | A | G | 0.0252071 | 0.00215485 | 1.31E-31 |  | 181 | 337,018 | -1.54E-06 | 5.74E-05 | 0.978599005 |
| rs9648476 | A | G | 0.0125013 | 0.00218542 | 1.06E-08 |  | 181 | 337,018 | 0.000104014 | 5.83E-05 | 0.074332716 |
| rs977474 | T | C | 0.0217813 | 0.00285559 | 2.39E-14 |  | 181 | 337,018 | -0.000112936 | 7.60E-05 | 0.137328919 |
| rs9937354 | A | G | -0.0140923 | 0.0021433 | 4.86E-11 |  | 181 | 337,018 | 1.09E-06 | 5.71E-05 | 0.984723994 |

SNP, single nucleotide polymorphism; EA, effect allele; OA, other allele; SE, standard error.

**Supplementary Table S2.2** SNPs from GWAS on tea intake and gout due to impairment of renal function

|  | | | **Exposure (Tea intake)** | | |  | **Outcome (Gout due to impairment of renal function)** | | | | |
| --- | --- | --- | --- | --- | --- | --- | --- | --- | --- | --- | --- |
| **SNP** | **EA** | **OA** | **β** | **SE** | ***P* value** |  | **Case** | **Control** | **β** | **SE** | ***P* value** |
| rs10741694 | C | T | 0.0150037 | 0.00219355 | 7.92E-12 |  | 242 | 368,788 | 0.133866 | 0.103009 | 0.193751 |
| rs10752269 | A | G | -0.0128727 | 0.00211975 | 1.26E-09 |  | 242 | 368,788 | -0.0186155 | 0.0952169 | 0.844997 |
| rs10764990 | A | G | -0.0121906 | 0.00216898 | 1.90E-08 |  | 242 | 368,788 | 0.078415 | 0.091471 | 0.391297 |
| rs11164870 | G | C | -0.0119604 | 0.00218232 | 4.24E-08 |  | 242 | 368,788 | -0.140226 | 0.0946288 | 0.138379 |
| rs1156588 | G | A | -0.015454 | 0.00260325 | 2.91E-09 |  | 242 | 368,788 | -0.0716878 | 0.108973 | 0.510633 |
| rs11587444 | G | A | 0.0140328 | 0.00217078 | 1.02E-10 |  | 242 | 368,788 | 0.0998085 | 0.0926307 | 0.281263 |
| rs12591786 | T | C | -0.0184399 | 0.00294243 | 3.68E-10 |  | 242 | 368,788 | 0.0692692 | 0.152476 | 0.649616 |
| rs13282783 | T | C | -0.0135837 | 0.00235432 | 7.94E-09 |  | 242 | 368,788 | 0.163746 | 0.093081 | 0.0785471 |
| rs132904 | C | G | 0.0166007 | 0.00255257 | 7.85E-11 |  | 242 | 368,788 | -0.158418 | 0.106844 | 0.138154 |
| rs141071726 | A | G | 0.0407321 | 0.00681204 | 2.24E-09 |  | 242 | 368,788 | -0.292693 | 0.381463 | 0.442908 |
| rs1453548 | A | T | -0.0133414 | 0.00224973 | 3.03E-09 |  | 242 | 368,788 | 0.0189275 | 0.0970342 | 0.845346 |
| rs149805207 | G | A | -0.0719337 | 0.0125823 | 1.08E-08 |  | 242 | 368,788 | 0.612513 | 0.33001 | 0.0634468 |
| rs17245213 | A | G | -0.0146481 | 0.00260905 | 1.97E-08 |  | 242 | 368,788 | -0.0840754 | 0.119887 | 0.483121 |
| rs17576658 | A | G | -0.0134812 | 0.00245655 | 4.07E-08 |  | 242 | 368,788 | 0.0997673 | 0.109474 | 0.362122 |
| rs17685 | A | G | 0.0230655 | 0.00236195 | 1.58E-22 |  | 242 | 368,788 | 0.044106 | 0.093957 | 0.638765 |
| rs2117137 | G | A | 0.0129948 | 0.0021557 | 1.66E-09 |  | 242 | 368,788 | -0.0327698 | 0.0914931 | 0.720218 |
| rs2273447 | T | A | 0.0174715 | 0.00263421 | 3.30E-11 |  | 242 | 368,788 | -0.122412 | 0.13294 | 0.357151 |
| rs2279844 | A | G | -0.0119879 | 0.00218318 | 4.00E-08 |  | 242 | 368,788 | -0.071244 | 0.0947035 | 0.45188 |
| rs2351187 | A | G | 0.0129023 | 0.0022823 | 1.57E-08 |  | 242 | 368,788 | -0.0892917 | 0.0983696 | 0.364028 |
| rs2472297 | T | C | 0.0533453 | 0.00240098 | 2.29E-109 |  | 242 | 368,788 | 0.00822051 | 0.104935 | 0.937558 |
| rs2478875 | G | A | 0.0218943 | 0.00261129 | 5.10E-17 |  | 242 | 368,788 | -0.056488 | 0.120971 | 0.640532 |
| rs2645929 | G | A | -0.0149842 | 0.0027166 | 3.47E-08 |  | 242 | 368,788 | -0.241075 | 0.126943 | 0.0575546 |
| rs34619 | A | G | 0.0117117 | 0.0021375 | 4.27E-08 |  | 242 | 368,788 | -0.173575 | 0.092662 | 0.0610394 |
| rs4410790 | C | T | 0.0405506 | 0.00219507 | 3.38E-76 |  | 242 | 368,788 | -0.0347245 | 0.0964851 | 0.718926 |
| rs4652274 | C | T | -0.0157568 | 0.0021744 | 4.28E-13 |  | 242 | 368,788 | 0.180051 | 0.0946293 | 0.0570808 |
| rs4808940 | G | C | 0.015123 | 0.00224718 | 1.70E-11 |  | 242 | 368,788 | -0.0174208 | 0.105253 | 0.86854 |
| rs4817505 | C | T | 0.015068 | 0.0021746 | 4.24E-12 |  | 242 | 368,788 | -0.0201131 | 0.0923798 | 0.827646 |
| rs56348300 | G | C | 0.0158824 | 0.00273191 | 6.11E-09 |  | 242 | 368,788 | 0.0290086 | 0.135495 | 0.830474 |
| rs57462170 | A | G | 0.0191505 | 0.00340563 | 1.87E-08 |  | 242 | 368,788 | -0.170316 | 0.131678 | 0.195863 |
| rs57631352 | G | A | -0.0131035 | 0.00232117 | 1.65E-08 |  | 242 | 368,788 | -0.063505 | 0.0962593 | 0.509428 |
| rs6829 | T | C | -0.0119163 | 0.00216546 | 3.74E-08 |  | 242 | 368,788 | 0.0861063 | 0.093007 | 0.354547 |
| rs713598 | G | C | 0.0133969 | 0.00215659 | 5.23E-10 |  | 242 | 368,788 | -0.023703 | 0.0955749 | 0.804131 |
| rs72797284 | G | A | -0.0171147 | 0.00238353 | 6.95E-13 |  | 242 | 368,788 | 0.249043 | 0.111973 | 0.0261403 |
| rs7757102 | G | A | -0.0118039 | 0.00213302 | 3.13E-08 |  | 242 | 368,788 | 0.00784366 | 0.091779 | 0.931894 |
| rs9302428 | G | C | 0.0122457 | 0.00220122 | 2.65E-08 |  | 242 | 368,788 | 0.000971325 | 0.0952291 | 0.991862 |
| rs9624470 | A | G | 0.0252071 | 0.00215485 | 1.31E-31 |  | 242 | 368,788 | -0.103921 | 0.0916146 | 0.256657 |
| rs9648476 | A | G | 0.0125013 | 0.00218542 | 1.06E-08 |  | 242 | 368,788 | -0.153747 | 0.0951381 | 0.106086 |
| rs977474 | T | C | 0.0217813 | 0.00285559 | 2.39E-14 |  | 242 | 368,788 | -0.0775698 | 0.161626 | 0.631274 |
| rs9937354 | A | G | -0.0140923 | 0.0021433 | 4.86E-11 |  | 242 | 368,788 | -0.050826 | 0.0918859 | 0.580166 |

SNP, single nucleotide polymorphism; EA, effect allele; OA, other allele; SE, standard error.

**Supplementary Table S2.3** SNPs from GWAS on tea intake and idiopathic gout

|  | | | **Exposure (Tea intake)** | | |  | **Outcome (Idiopathic gout)** | | | | |
| --- | --- | --- | --- | --- | --- | --- | --- | --- | --- | --- | --- |
| **SNP** | **EA** | **OA** | **β** | **SE** | ***P* value** |  | **Case** | **Control** | **β** | **SE** | ***P* value** |
| rs10741694 | C | T | 0.0150037 | -0.0130097 | 7.92E-12 |  | 2140 | 368788 | -0.0130097 | 0.0349609 | 0.709803 |
| rs10752269 | A | G | -0.0128727 | 0.0189943 | 1.26E-09 |  | 2140 | 368788 | 0.0189943 | 0.0322746 | 0.556183 |
| rs10764990 | A | G | -0.0121906 | 0.0447531 | 1.90E-08 |  | 2140 | 368788 | 0.0447531 | 0.0309455 | 0.148124 |
| rs11164870 | G | C | -0.0119604 | 0.0376379 | 4.24E-08 |  | 2140 | 368788 | 0.0376379 | 0.0320472 | 0.240214 |
| rs1156588 | G | A | -0.015454 | -0.00663251 | 2.91E-09 |  | 2140 | 368788 | -0.00663251 | 0.0366391 | 0.856349 |
| rs11587444 | G | A | 0.0140328 | 0.0156114 | 1.02E-10 |  | 2140 | 368788 | 0.0156114 | 0.0313705 | 0.618734 |
| rs12591786 | T | C | -0.0184399 | -0.0163989 | 3.68E-10 |  | 2140 | 368788 | -0.0163989 | 0.0518908 | 0.751981 |
| rs13282783 | T | C | -0.0135837 | -0.0205099 | 7.94E-09 |  | 2140 | 368788 | -0.0205099 | 0.0316007 | 0.516316 |
| rs132904 | C | G | 0.0166007 | 0.0167878 | 7.85E-11 |  | 2140 | 368788 | 0.0167878 | 0.0361007 | 0.641913 |
| rs141071726 | A | G | 0.0407321 | -0.0333623 | 2.24E-09 |  | 2140 | 368788 | -0.0333623 | 0.128414 | 0.795016 |
| rs1453548 | A | T | -0.0133414 | -0.0362032 | 3.03E-09 |  | 2140 | 368788 | -0.0362032 | 0.0327517 | 0.268994 |
| rs149805207 | G | A | -0.0719337 | 0.0940751 | 1.08E-08 |  | 2140 | 368788 | 0.0940751 | 0.113793 | 0.408396 |
| rs17245213 | A | G | -0.0146481 | 0.0689879 | 1.97E-08 |  | 2140 | 368788 | 0.0689879 | 0.0406498 | 0.0896726 |
| rs17576658 | A | G | -0.0134812 | -0.0424369 | 4.07E-08 |  | 2140 | 368788 | -0.0424369 | 0.0370478 | 0.252017 |
| rs17685 | A | G | 0.0230655 | -0.0140705 | 1.58E-22 |  | 2140 | 368788 | -0.0140705 | 0.0317507 | 0.657652 |
| rs2273447 | T | A | 0.0174715 | 0.0558949 | 3.30E-11 |  | 2140 | 368788 | 0.0558949 | 0.0453784 | 0.218042 |
| rs2279844 | A | G | -0.0119879 | -0.00965401 | 4.00E-08 |  | 2140 | 368788 | -0.00965401 | 0.0319827 | 0.762765 |
| rs2351187 | A | G | 0.0129023 | -0.0247725 | 1.57E-08 |  | 2140 | 368788 | -0.0247725 | 0.033303 | 0.456966 |
| rs2478875 | G | A | 0.0218943 | 0.0376274 | 5.10E-17 |  | 2140 | 368788 | 0.0376274 | 0.0409882 | 0.358615 |
| rs2645929 | G | A | -0.0149842 | 0.00338164 | 3.47E-08 |  | 2140 | 368788 | 0.00338164 | 0.0433377 | 0.937804 |
| rs4410790 | C | T | 0.0405506 | 0.00232986 | 3.38E-76 |  | 2140 | 368788 | 0.00232986 | 0.0327138 | 0.943223 |
| rs4808940 | G | C | 0.015123 | -0.00380858 | 1.70E-11 |  | 2140 | 368788 | -0.00380858 | 0.0358743 | 0.915452 |
| rs4817505 | C | T | 0.015068 | -0.00898842 | 4.24E-12 |  | 2140 | 368788 | -0.00898842 | 0.0312923 | 0.773929 |
| rs56348300 | G | C | 0.0158824 | 0.0317356 | 6.11E-09 |  | 2140 | 368788 | 0.0317356 | 0.0462179 | 0.492302 |
| rs57631352 | G | A | -0.0131035 | -0.00742546 | 1.65E-08 |  | 2140 | 368788 | -0.00742546 | 0.0326413 | 0.820045 |
| rs6829 | T | C | -0.0119163 | 0.0746201 | 3.74E-08 |  | 2140 | 368788 | 0.0746201 | 0.0314938 | 0.0178189 |
| rs713598 | G | C | 0.0133969 | 0.0423198 | 5.23E-10 |  | 2140 | 368788 | 0.0423198 | 0.0324002 | 0.191499 |
| rs72797284 | G | A | -0.0171147 | 0.0585605 | 6.95E-13 |  | 2140 | 368788 | 0.0585605 | 0.03794 | 0.122709 |
| rs7757102 | G | A | -0.0118039 | 0.0270474 | 3.13E-08 |  | 2140 | 368788 | 0.0270474 | 0.0310724 | 0.384047 |
| rs9302428 | G | C | 0.0122457 | -0.0743162 | 2.65E-08 |  | 2140 | 368788 | -0.0743162 | 0.0321434 | 0.0207764 |
| rs9624470 | A | G | 0.0252071 | -0.0765355 | 1.31E-31 |  | 2140 | 368788 | -0.0765355 | 0.0310187 | 0.0136097 |
| rs9648476 | A | G | 0.0125013 | -0.00851353 | 1.06E-08 |  | 2140 | 368788 | -0.00851353 | 0.0320546 | 0.790551 |
| rs977474 | T | C | 0.0217813 | -0.0125482 | 2.39E-14 |  | 2140 | 368788 | -0.0125482 | 0.0555 | 0.821128 |
| rs9937354 | A | G | -0.0140923 | -0.0125462 | 4.86E-11 |  | 2140 | 368788 | -0.0125462 | 0.0311036 | 0.686678 |

SNP, single nucleotide polymorphism; EA, effect allele; OA, other allele; SE, standard error.

**Supplementary Table S2.4** SNPs from GWAS on tea intake and uric acid

|  | | | **Exposure (Tea intake)** | | |  | **Outcome (Uric acid)** | | | |
| --- | --- | --- | --- | --- | --- | --- | --- | --- | --- | --- |
| **SNP** | **EA** | **OA** | **β** | **SE** | ***P* value** |  | **[Sample size](https://gwas.mrcieu.ac.uk/datasets/?trait__icontains=uric+acid&sort=sample_size)** | **β** | **SE** | ***P* value** |
| rs10741694 | C | T | 0.0150037 | 0.00219355 | 7.92E-12 |  | 109,029 | -0.006745 | 0.005046 | 0.181300082 |
| rs10752269 | A | G | -0.0128727 | 0.00211975 | 1.26E-09 |  | 109,029 | 0.00301 | 0.004414 | 0.495200415 |
| rs10764990 | A | G | -0.0121906 | 0.00216898 | 1.90E-08 |  | 109,029 | 0.002535 | 0.004223 | 0.54829969 |
| rs1156588 | G | A | -0.015454 | 0.00260325 | 2.91E-09 |  | 109,029 | -0.003238 | 0.005105 | 0.525800506 |
| rs11587444 | G | A | 0.0140328 | 0.00217078 | 1.02E-10 |  | 109,029 | -0.008629 | 0.004297 | 0.04463957 |
| rs12591786 | T | C | -0.0184399 | 0.00294243 | 3.68E-10 |  | 109,029 | 0.001313 | 0.007334 | 0.858000024 |
| rs13282783 | T | C | -0.0135837 | 0.00235432 | 7.94E-09 |  | 109,029 | -0.001893 | 0.004307 | 0.660299548 |
| rs1453548 | A | T | -0.0133414 | 0.00224973 | 3.03E-09 |  | 109,029 | 0.005383 | 0.00464 | 0.245999939 |
| rs17685 | A | G | 0.0230655 | 0.00236195 | 1.58E-22 |  | 109,029 | -0.006227 | 0.004377 | 0.154800016 |
| rs2117137 | G | A | 0.0129948 | 0.0021557 | 1.66E-09 |  | 109,029 | -0.00755 | 0.006606 | 0.253099915 |
| rs2273447 | T | A | 0.0174715 | 0.00263421 | 3.30E-11 |  | 109,029 | -0.001382 | 0.01048 | 0.895100088 |
| rs2279844 | A | G | -0.0119879 | 0.00218318 | 4.00E-08 |  | 109,029 | 0.005826 | 0.004208 | 0.166199993 |
| rs2351187 | A | G | 0.0129023 | 0.0022823 | 1.57E-08 |  | 109,029 | 0.01047 | 0.02804 | 0.708800475 |
| rs2645929 | G | A | -0.0149842 | 0.0027166 | 3.47E-08 |  | 109,029 | -0.0009029 | 0.006256 | 0.885199989 |
| rs34619 | A | G | 0.0117117 | 0.0021375 | 4.27E-08 |  | 109,029 | -0.005191 | 0.004958 | 0.295099858 |
| rs4410790 | C | T | 0.0405506 | 0.00219507 | 3.38E-76 |  | 109,029 | -0.0002627 | 0.004478 | 0.953199926 |
| rs4652274 | C | T | -0.0157568 | 0.0021744 | 4.28E-13 |  | 109,029 | -0.007707 | 0.00455 | 0.090300468 |
| rs4817505 | C | T | 0.015068 | 0.0021746 | 4.24E-12 |  | 109,029 | -0.002511 | 0.004219 | 0.55170002 |
| rs57462170 | A | G | 0.0191505 | 0.00340563 | 1.87E-08 |  | 109,029 | 0.005417 | 0.005955 | 0.362900027 |
| rs57631352 | G | A | -0.0131035 | 0.00232117 | 1.65E-08 |  | 109,029 | 0.001661 | 0.004198 | 0.692399906 |
| rs6829 | T | C | -0.0119163 | 0.00216546 | 3.74E-08 |  | 109,029 | 0.003802 | 0.01103 | 0.730299494 |
| rs72797284 | G | A | -0.0171147 | 0.00238353 | 6.95E-13 |  | 109,029 | 0.01296 | 0.01377 | 0.346499809 |
| rs9302428 | G | C | 0.0122457 | 0.00220122 | 2.65E-08 |  | 109,029 | 0.0008835 | 0.008018 | 0.912300053 |
| rs9624470 | A | G | 0.0252071 | 0.00215485 | 1.31E-31 |  | 109,029 | -0.004685 | 0.004807 | 0.329800265 |
| rs9648476 | A | G | 0.0125013 | 0.00218542 | 1.06E-08 |  | 109,029 | 0.02371 | 0.01473 | 0.107599933 |
| rs977474 | T | C | 0.0217813 | 0.00285559 | 2.39E-14 |  | 109,029 | 0.02257 | 0.0173 | 0.19219983 |
| rs9937354 | A | G | -0.0140923 | 0.0021433 | 4.86E-11 |  | 109,029 | 0.001689 | 0.005019 | 0.736400373 |

SNP, single nucleotide polymorphism; EA, effect allele; OA, other allele; SE, standard error.

**Supplementary Table S3** The results of MR-Egger intercept analysis

| **Exposure** | **Outcome** | **Egger_intercept** | **SE** | ***P* value** |
| --- | --- | --- | --- | --- |
| Tea intake | Gout | 2.51E-05 | 2.70E-05 | 0.358915546 |
| Tea intake | Gout due to impairment of renal function | -0.007827003 | 0.037694992 | 0.836648424 |
| Tea intake | Idiopathic gout | -0.005797004 | 0.0165544 | 0.728498862 |
| Tea intake | Uric acid | -0.003184141 | 0.002753719 | 0.25848681 |

**Supplementary Table S4** The results of heterogeneity analysis

| **Exposure** | **Outcome** | **Method** | **Q** | **Q_df** | **Q_*P* val** |
| --- | --- | --- | --- | --- | --- |
| Tea intake | Gout | MR Egger | 51.59228765 | 37 | 0.056019933 |
| Tea intake | Gout | Inverse variance weighted | 52.79567466 | 38 | 0.055826381 |
| Tea intake | Gout due to impairment of renal function | MR Egger | 39.20524252 | 37 | 0.371209282 |
| Tea intake | Gout due to impairment of renal function | Inverse variance weighted | 39.25092673 | 38 | 0.413595318 |
| Tea intake | Idiopathic gout | MR Egger | 34.54681303 | 32 | 0.347087071 |
| Tea intake | Idiopathic gout | Inverse variance weighted | 34.67919788 | 33 | 0.387773585 |
| Tea intake | Uric acid | MR Egger | 21.29651156 | 25 | 0.675964924 |
| Tea intake | Uric acid | Inverse variance weighted | 22.63355445 | 26 | 0.653585625 |
